# Establishment of an efficient cotton root protoplast isolation protocol suitable for single-cell RNA sequencing and transient gene expression analysis

**DOI:** 10.1186/s13007-023-00983-6

**Published:** 2023-01-18

**Authors:** Ke Zhang, Shanhe Liu, Yunze Fu, Zixuan Wang, Xiubo Yang, Wenjing Li, Caihua Zhang, Dongmei Zhang, Jun Li

**Affiliations:** 1 State Key Laboratory of North China Crop Improvement and Regulation, College of Agronomy, Hebei Agricultural University, Baoding, 071001 China; 2 Hebei Key Laboratory of Plant Physiology and Molecular Pathology, College of Life Sciences, Hebei Agricultural University, Baoding, 071001 China; 3 Key Laboratory of Crop Growth Regulation of Hebei Province, Hebei Agricultural University, Baoding, 071001 China; 4 Key Laboratory for Crop Germplasm Resources of Hebei, Hebei Agricultural University, Baoding, 071001 China

**Keywords:** Protoplast isolation, PEG-mediated transfection, scRNA-seq, Genome editing, Cotton

## Abstract

**Background:**

Cotton has tremendous economic value worldwide; however, its allopolyploid nature and time-consuming transformation methods have hampered the development of cotton functional genomics. The protoplast system has proven to be an important and versatile tool for functional genomics, tissue-specific marker gene identification, tracking developmental trajectories, and genome editing in plants. Nevertheless, the isolation of abundant viable protoplasts suitable for single-cell RNA sequencing (scRNA-seq) and genome editing remains a challenge in cotton.

**Results:**

We established an efficient transient gene expression system using protoplasts isolated from cotton taproots. The system enables the isolation of large numbers of viable protoplasts and uses an optimized PEG-mediated transfection protocol. The highest yield (3.55 × 10^5^/g) and viability (93.3%) of protoplasts were obtained from cotton roots grown in hydroponics for 72 h. The protoplasts isolated were suitable for scRNA-seq. The highest transfection efficiency (80%) was achieved when protoplasts were isolated as described above and transfected with 20 μg of plasmid for 20 min in a solution containing 200 mM Ca^2+^. Our protoplast-based transient expression system is suitable for various applications, including validation the efficiency of CRISPR vectors, protein subcellular localization analysis, and protein–protein interaction studies.

**Conclusions:**

The protoplast isolation and transfection protocol developed in this study is stable, versatile, and time-saving. It will accelerate functional genomics and molecular breeding in cotton.

**Supplementary Information:**

The online version contains supplementary material available at 10.1186/s13007-023-00983-6.

## Background

As the main fiber crop in China, cotton is an essential textile raw material and national strategic material; thus, it has close ties to the national economy and people’s livelihood [1]. Improvements in cotton breeding and growth control technologies will ensure the rapid development of the cotton planting industry. Functional genomics provides a molecular basis for crop improvement and is an effective way to promote efficient and precise breeding. The recent sequencing and assembly of the cotton genome has opened the door to the systematic study of gene function [2–4]. The emergence of modern biotechnologies, including single-cell RNA sequencing (scRNA-seq) and genome editing, will promote the development of breeding strategies and basic research in cotton [5–8].

scRNA-seq is a rapidly evolving and increasingly mature technology that shows great power in resolving cell responses to developmental and environmental cues [9]. It offers distinct advantages in the detection of rare cell types/states, the mining of detailed spatio-temporal transcript information as well as tissue- and developmental stage-specific marker genes, and the resolution of developmental trajectories in complex tissue [10, 11]. Unlike animal cells, plant cells are surrounded by cell walls. Thus, the prerequisite for applying scRNA-seq to plants is dissociating the cell wall to isolate protoplasts.

The developmental stage of the materials used is critical for successful protoplast isolation [12]. During seed germination, the radicle breaks through the seed coat to form a primary root and begin primary growth. Gradually, morphogenesis of the root tip, including the root cap, meristematic zone, elongation zone, and mature zone, is completed. About 4 days after germination, lateral roots begin to emerge from the base of the primary root in ascending order, indicating the beginning of secondary growth. Once the primary structure is formed, many cell types exist in the root, the tissue is youthful and tender, and the cell walls are thin, making it the best time to isolate protoplasts for use in scRNA-seq [13–15].

Cell number, cell size, and protoplast viability impact the quality of scRNA-seq data. A sufficient number of cells is vital to ensure the capture of rare cells in tissues. Usually for tissues with a simple structure, such as *Arabidopsis* roots, 10,000 cells are sufficient, while for tissues with a complex structure, additional cells are needed [16]. For droplet-based techniques, tiny cells are more likely to be captured due to a size-biased effect. Large cells may block the pipeline; therefore, 10 × Genomics’ commonly used Chromium platform requires that the cell size cannot exceed 40–50 μm [16]. The percent viability is commonly required to be  > 80% according to 10 × Genomics. Additionally, since the Ca^2+^ and Mg^2+^ contained in the enzyme and MMG solutions can interfere with subsequent reverse transcription [17] and cause intercellular adhesion or clumping, the protoplasts used for scRNA-seq must generally be resuspended in mannitol to maintain the correct osmotic pressure and viability.

Genome editing, or genome engineering, allows the precise modification of specific genomic sites to generate targeted mutants [18]. Breeding approaches based on genome editing have become the fourth major type of breeding technology after cross-breeding, mutation breeding, and transgenic breeding; together, these methods have given rise to precision breeding techniques that are defining the next-generation of plant breeding [5]. However, to apply gene editing technology to certain crops, especially those are difficult or lengthy to transform, it is necessary not only to optimize the genome editing efficiency, plant cell delivery, and regeneration system, but also to develop efficient protoplast transfection methods to verify the activity of CRISPR vectors [19–21].

Taken together, the developmental stage of the materials, yield, and viability are important factors in protoplast isolation as they will impact the quality of the scRNA-seq data and the transient expression system for various applications. In this study, we determined the suitable root age for protoplast isolation from taproots, defined the digestion time, and adjusted the preparation scheme to produce protoplasts that are usable for scRNA-seq in terms of cell number, viability, size, and purity. Furthermore, we formulated an efficient protoplast transient expression system by optimizing the Ca^2+^ concentration, plasmid concentration, and incubation time. Our protoplast isolation scheme can be used for scRNA-seq, and the optimized transient transformation system is suitable both for the verification of CRISPR vectors and for use in gene function studies, including studies of protein subcellular localization and protein–protein interactions.

## Methods

### Plant materials and growth conditions

Delinted cotton (*Gossypium hirsutum* cv. ND601) seeds were surface-sterilized in 75% ethanol for 10 min followed by 3% sodium hypochlorite treatment for 10 min and rinsed five times in sterile water. After soaking the seeds in sterile water for 7 h, they were placed between two layers of wet towels to retain moisture and air, and then placed in a 25 °C incubator for about 36 h. When the length of the radicle root reached about 1 cm, seeds showing normal germination were transferred to a hydroponic container for culture at 28/25 °C (day/night), with a 16 h/8 h (day/night) photoperiod (600 μmol·m^−2^·s^−1^).

### Isolation of protoplasts from cotton taproots

Protoplasts were isolated following the methods of Yoo [22], Li [23], and Wu [24] with modifications as shown below.

1. Taproots of cotton plants grown in hydroponics for 72 h after germination were used to isolate protoplasts.

Note: The timing of hydroponics is critical; we found that taproots after 65–75 h of hydroponic culture were suitable for protoplast isolation, whereas hydroponic culture for < 48 h resulted in increased tissue fragments in the cell suspension. However, if the time exceeded 96 h, the cell harvest rate was significantly reduced.

(2) Tapoots from 25–50 seedlings were cut into 0.5–1-mm slices and dipped in 10 ml of enzyme solution in a 50 ml conical flask.

Note 1: The enzyme solution was freshly prepared. It contained 1.5% (w/v) Cellulase R10 (Yakult, Tokyo, Japan), 0.75% (w/v) Macerozyme R10 (Yakult), 0.4 M mannitol, 20 mM KCl, and 20 mM MES (pH 5.7). Once prepared, the solution was warmed at 55 °C for 10 min. Upon cooling to room temperature, 10 mM CaCl_2_ and 0.1% bovine serum albumin were added, and the solution was filtered with a 0.45 μm Millipore filter (MilliporeSigma, Burlington, MA, USA).

Note 2: The slices must be thoroughly immersed in the enzyme solution. Sample cutting can be performed on plastic cultures or seed germination pouches (CYG-38LB; PhytoTC, Shanghai, China). In our experience, cutting 5–7 roots together is efficient and does not affect the isolation process. Slice the sample using a Gillette razor blade (Boston, MA, USA) from one side to the other. The cutting speed should not be overly rapid. The thickness is appropriate when the slice is translucent. It is necessary to switch the blade during slicing. Normally, for 25 roots you will use two blades.

(3) Samples were incubated for 3 h in the enzyme solution with shaking on a shaker at a speed of 40–50 rpm at 25 °C in the dark.

Note: The number of protoplasts released can be estimated based on the turbidity of the enzyme solution; however, microscopic examination is the most accurate method.

(4) An equal volume of W5 solution [154 mM NaCl, 125 mM CaCl_2_, 5 mM KCl, and 2 mM MES (pH 5.7)] was added to the enzyme mixture, which was shaken vigorously for 10 s to release the protoplasts and then filtered using four layers of Miracloth (MilliporeSigma).

Note: To maximize the protoplast yield, gently transfer the tissue residue back to the conical flask and add 10 ml of W5 solution. Incubate the mixture for 1 h on a shaker at a speed of 40–50 rpm at 25 °C in the dark, and then proceed to step (4).

(5) The filtrate was filtered into a 50 ml centrifuge tube using a 40 μm cell strainer.

Note: In our experience, the range of cell sizes in root tissue is relatively large. Due to technical limitations, the cell size for scRNA-seq cannot be > 40 μm. In some cases, it is necessary to use a 30 μm cell strainer. The strainer should be moistened with W5 solution before use. We suggest using a round-bottomed tube throughout the experiment.

(6) The mixture was centrifuged horizontally at 25 °C at 100 g for 5 min to pellet the protoplasts. The supernatant was discarded gently without disturbing the pellet.

Note: We recommend setting the centrifuge’s accelerate and decelerate controls to 1 or using the soft key. A centrifuge with a swinging-bucket rotor is well-suited for protoplast collection.

(7) The protoplasts were resuspended in 5 ml of pre-chilled W5 solution. The suspension was kept on ice for 30–60 min.

Note: If the protoplasts are to be used for scRNA-seq, the cell suspension should not contain MgCl_2_ or CaCl_2_. Therefore, the protoplasts must be resuspended in 5 ml of pre-chilled 0.5 M mannitol.

(8) The supernatant was carefully removed without touching the protoplast pellet. The protoplasts were then resuspended to a final concentration of 1 × 10^6^ with MMG solution (4 mM MES, 0.4 M mannitol, and 15 mM MgCl_2_). The viability was determined using 0.01% (w/v) FDA staining [25].

Note: If the protoplasts are to be used for scRNA-seq, they should be resuspended in 0.5 M mannitol. If LSCs (≥ 40 um) are observed by microscopic examination, a 30 μm cell strainer should be used for filtration in step (5). All pipette tips used for protoplast isolation should be cut with scissors as the protoplasts are extremely fragile.

### Vector construction and plasmid preparation

We used the CRISPR/Cas9 system as described previously [26]. One reported active target site [27] was used to test the protoplast transient expression system. Two sites targeting *PDS* and *CLA* were also designed, respectively. Pairs of oligonucleotides including the targeting sequences (Additional file 1) were synthesized, annealed, and cloned into *Bsa*I-digested *pKSE401* [26]. The targeting vectors were verified by sequencing and extracted using a Fastpure DNA Isolation Mini Kit (Vazyme Biotech, Shanghai, China). The *35S-GFP* sequence, amplified from *pBI221-CaMV35S-GFP* [28], was cloned into *pEASY-Blunt* (TransGen Biotech, Beijing, China) to obtain *pEASY-35S:GFP*. *pUC18-Man49-mCherry* was used for protein subcellular localization analysis [29, 30]. For bimolecular fluorescence complementation (BiFC) assays, the coding sequences of *GhBIN2* and *GhBZR3* were cloned into the BiFC expression vectors *p326-YFP*^*N*^ and *p326-YFP*^*C*^ [31], respectively, to obtain *p326-GhBIN2-YFP*^*N*^ and *p326-GhBZR3-YFP*^*C*^. The plasmids were extracted using a commercial kit (Wizard^®^ Plus Midipreps DNA Purification System; Promega Biotech, Beijing, China) to yield quality DNAs (> 1,000 ng/μl).

### PEG-mediated protoplast transfection

PEG-mediated protoplast transfection was carried out as described previously with modifications [22]. For each transformation, 20 μl of DNA (10–20 μg of plasmid) were gently mixed with 200 μl of protoplasts in a 2-ml microfuge tube. Then, 220 μl of freshly prepared PEG solution [40% (w/v) PEG4000, 200 mM mannitol, and 50–300 mM CaCl_2_] were added and mixed completely by gently tapping the tube. The transfection mixture was incubated at 25 °C in the dark for different time periods (5, 10, 15, 20, or 25 min). It was stopped by adding 880 μl of W5 solution and mixed well by gently inverting the tube. After centrifugation at 100 g for 3 min, the supernatant was removed and the protoplasts were resuspended in 1.5 ml of W5 solution and then transferred to a 2 ml tube. For transient expression of the genome editing reagents or proteins (genes), transfected protoplasts were incubated for 12–48 h at 25 °C in darkness. Based on the experimental purpose, the materials used in the transfection system may be scaled up or down.

### Verification of CRISPR/Cas9-mediated mutations in cotton protoplasts

A PCR/restriction enzyme (RE) assay was used to assess the activity of the targeting vectors. Genomic DNA was extracted from pooled protoplasts transformed with the targeting vectors, and the sequence (~ 700 bp) encompassing the CRISPR target site was amplified by PCR using a high-fidelity DNA polymerase. The amplicon was then digested with the appropriate RE and analyzed by gel electrophoresis. Mutations induced by non-homologous end joining (NHEJ) were resistant to RE digestion, resulting in an uncleaved band. Uncleaved bands were purified and then cloned into the cloning vector T-Blunt. The resulting transformants were identified by colony PCR. Subsequently, positive clones were sequenced using T7 primer to obtain the mutated sequences.

### RNA extraction and gene expression analysis

Total RNA was isolated from roots tissue or protoplasts using TransZol reagent (TransGen Biotech); genomic DNA contamination was eliminated with DNase I (Roche). M-MLV reverse transcriptase (Thermo Scientific) was used for first-strand cDNA synthesis. Real-time PCR was conducted in technical triplicates using SYBR Green PCR master mix (DBI Bioscience). *GhACTIN14* was used as the reference gene. Three to four biological replicates were performed; the results were analyzed with SPSS statistics 17.0 (IBM).

### Microscopy

The protoplast yield was determined using a standard hemocytometer and light microscopy. The plasmid *pEASY-35S:GFP* was used to calculate the transformation efficiency of protoplasts and assess protein subcellular localization. GFP fluorescence was observed under a Nikon Ti-2U Fluorescence Microscope (Nikon Corp., Tokyo, Japan). The transformation efficiency (%) was calculated by dividing the protoplast number with bright green fluorescence by the total protoplast number. Images were acquired with an Olympus FV10i Confocal Microscope (Olympus Corp., Tokyo, Japan) using excitation wavelengths of 488 nm (GFP and YFP) and 561 nm (mCherry).

## Results

### Establishment of an efficient method for cotton root protoplast isolation

**Fig. 1:**
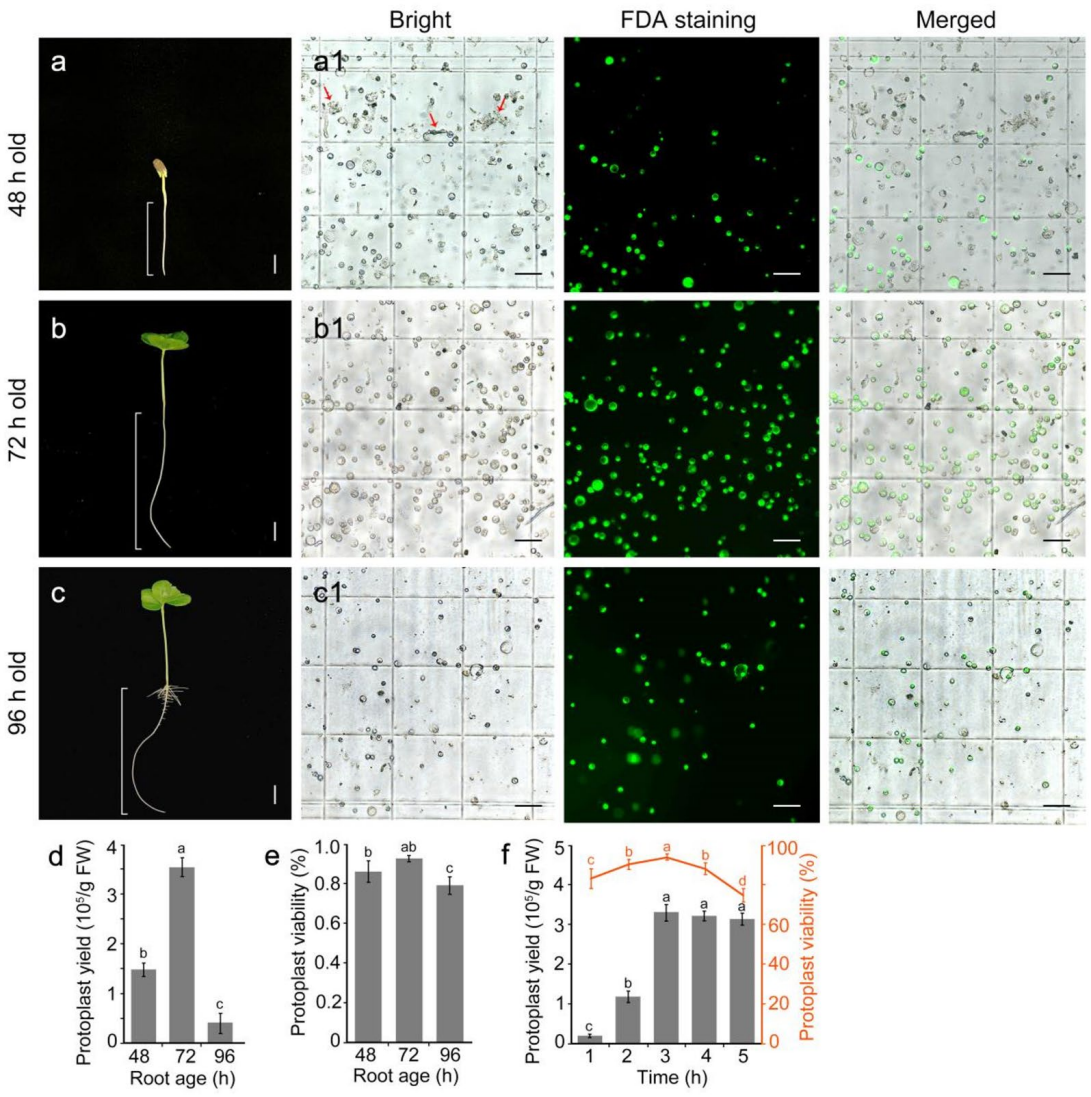
Establishment of a method for cotton root protoplast isolation. **a–c** Cotton seedlings were grown hydroponically for 48, 72, or 96 h after germination. White lines mark the sections of root tissue used to isolate protoplasts. Bars = 1 cm. **a–c** FDA staining of protoplasts from roots of different ages (48, 72, and 96 h). Red arrows mark tissue debris. Bars = 100 μm. **d** Protoplast yield as affected by root age (48, 72, and 96 h). **e** Protoplast viability as affected by root age (48, 72, and 96 h). **f** Protoplast yield and protoplast viability as affected by digestion time (1, 2, 3, 4, and 5 h)

To develop a protoplast isolation method suitable for single-cell sequencing and transient transfection, we considered methods reported previously for *Arabidopsis* and rice [22, 32]. We focused on optimizing root selection by age, the cell wall digestion time, and the mannitol concentration used to resuspended cells to meet scRNA-seq requirements. To determine the most suitable root age for protoplast isolation, we isolated and compared protoplasts from taproots of cotton grown in hydroponics for 48, 72, or 96 h (Fig. 1a–c). Roots of seedlings grown in hydroponics for 72 h had the highest protoplast yield (3.55 × 10^5^ protoplasts/g fresh weight [FW]) (Fig. 1d). FDA staining and Trypan blue staining revealed that 93.3% and 92.1% of the protoplasts were viable, respectively (Fig. 1b–e, and Additional file 2). The protoplast yield from roots grown in hydroponics for 48 h was 1.49 × 10^5^ protoplasts/g FW (Fig. 1d). FDA and Trypan blue staining showed that 86.6% and 70.2% of the protoplasts were viable; however, the impurity level was significantly higher than that in the other two groups (Fig. 1b–e). The lowest cell yield was 0.42 × 10^5^ protoplasts/g FW (Fig. 1d) and the percent cell viability was 79.7% (FDA) and 77.4% (Trypan blue) after 96 h of hydroponic culture (Fig. 1c–e). Therefore, cotton roots grown in hydroponics for 72 h were deemed most suitable for protoplast isolation.

The duration of enzyme incubation affected both the yield and viability of protoplasts. The testing of various enzyme digestion periods revealed that digestion for 3 h produced the highest protoplast isolation efficiency and viability (Fig. 1f and Additional file 3). Typically, scRNA-seq requires 1 × 10^5^–5 × 10^5^ prepared cells, and a loading capacity of 0.5 × 10^4^–2 × 10^4^ cells per channel; thus, the cell concentration should be 700–2,000 cells/μl, and the percent cell viability should be  > 80%, and preferably  > 90% (Trypan blue staining is recommended). To evaluate the efficiency of gene editing, 1 × 10^5^ cells per sample are usually required for transient transformation experiments, and cell viability  > 85% is preferred. Thus, protoplasts isolated from about 1 g of 72-h-old taproots (about 30 roots) incubated in enzyme solution for 3 h are sufficient for single-cell sequencing and for transformation experiments with 3–4 samples.

### A cotton root protoplast preparation protocol suitable for scRNA-seq

**Fig. 2:**
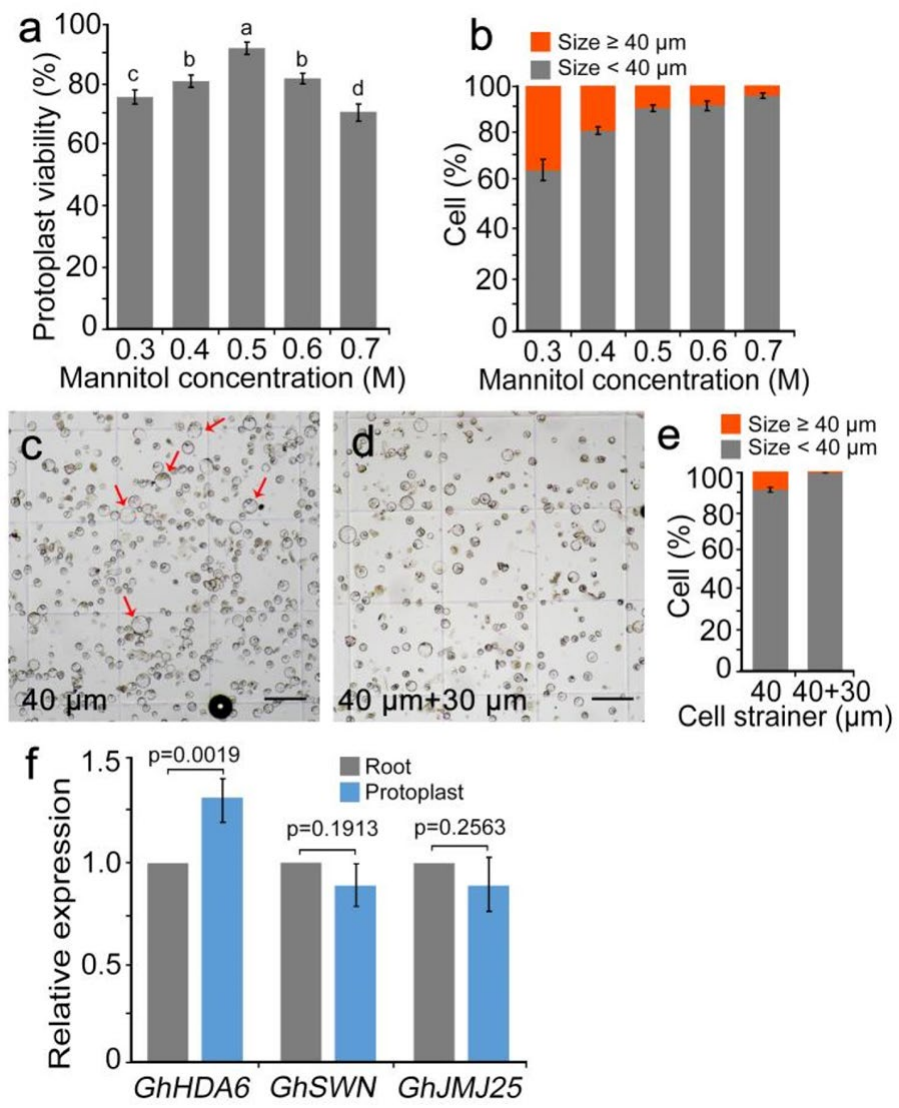
Optimization of the protoplast isolation protocol for single-cell sequencing. **a** Effects of using different mannitol concentrations in the resuspending solution on protoplast activity (0.3, 0.4, 0.5, 0.6, and 0.7 M). **b** The proportion of LSCs (≥ 40 μm) observed at different mannitol concentrations. **c** and **d** Compared with using a 40 μm cell strainer (c), LSCs were significantly reduced in number by using 40 and 30 μm cell strainers, successively (d). Red arrows mark LSCs. Bars = 100 μm. **e** Effect of different filtration methods on the proportion of LSCs in the cell suspension. **f** Expression analysis of genes involved in epigenetic modification between digested protoplasts and unprocessed root tissue

To exclude the influence of Ca^2+^ and Mg^2+^ introduced during cell preparation on subsequent scRNA-seq, it is necessary to resuspend the protoplasts in mannitol. Different concentrations of mannitol produce different penetrant pressures, which may affect cell viability and size. Therefore, to determine the optimal concentration of mannitol, we carried out gradient screening using concentrations of 0.3, 0.4, 0.5, 0.6, and 0.7 M. Protoplasts resuspended in 0.5 M mannitol had the highest viability (92.7%) (Fig. 2a and Additional file 4). Furthermore, since droplet-based techniques require that the cell diameter not exceed 40–50 μm, we defined cells with a diameter  ≥ 40 μm as LSCs. The proportion of LSCs increased significantly as the mannitol concentration decreased. The proportion of LSCs was the highest using 0.3 M mannitol (27.7%), but it was only 4.0% with 0.7 M mannitol, 11.3% with 0.5 M mannitol, and 10.2% with 0.6 M mannitol (Fig. 2b). Given these results, using a 40 μm cell strainer may not be adequate to remove all large cells, so we tried subsequently filtering the cell suspension with a 30 μm cell strainer to reduce the proportion of LSCs (Fig. 2c, d). The proportion of LSCs could be reduced  < 1.0% by filtering the cell suspension through a 40 μm cell strainer followed by a 30 μm cell strainer (Fig. 2e).

Although RNA-seq can be performed to identify and filter out genes induced during cell preparation from scRNA-seq data, it is necessary to detect changes in the expression of development-related genes that are regulated by epigenetic modification. Therefore, we examined the transcript levels of *GhHDA6*, *GhHDA19*, *GhSWN*, and *GhJMJ25*, which encode key factors in histone modification [33]. No significant changes in the expression of these genes were found between undigested roots and protoplasts (Fig. 2f), suggesting that our protoplast isolation protocol had only a slight effect on epigenetic remodeling.

### Optimization of a PEG-mediated protoplast transformation system in cotton

To test whether protoplasts isolated using the above protocol are suitable for transient gene expression, PEG-Ca^2+^ transfection of plasmids was carried out as described previously [22]. The vector *pEASY-35S:GFP* (5.8 kb) was used to calculate the transformation efficiency and determine the optimal DNA transfection conditions. The transformation efficiency was only ~ 20% in protoplasts isolated from roots at 72 h of growth (Additional file 5), indicating a need for further optimization of the protocol.

**Fig. 3:**
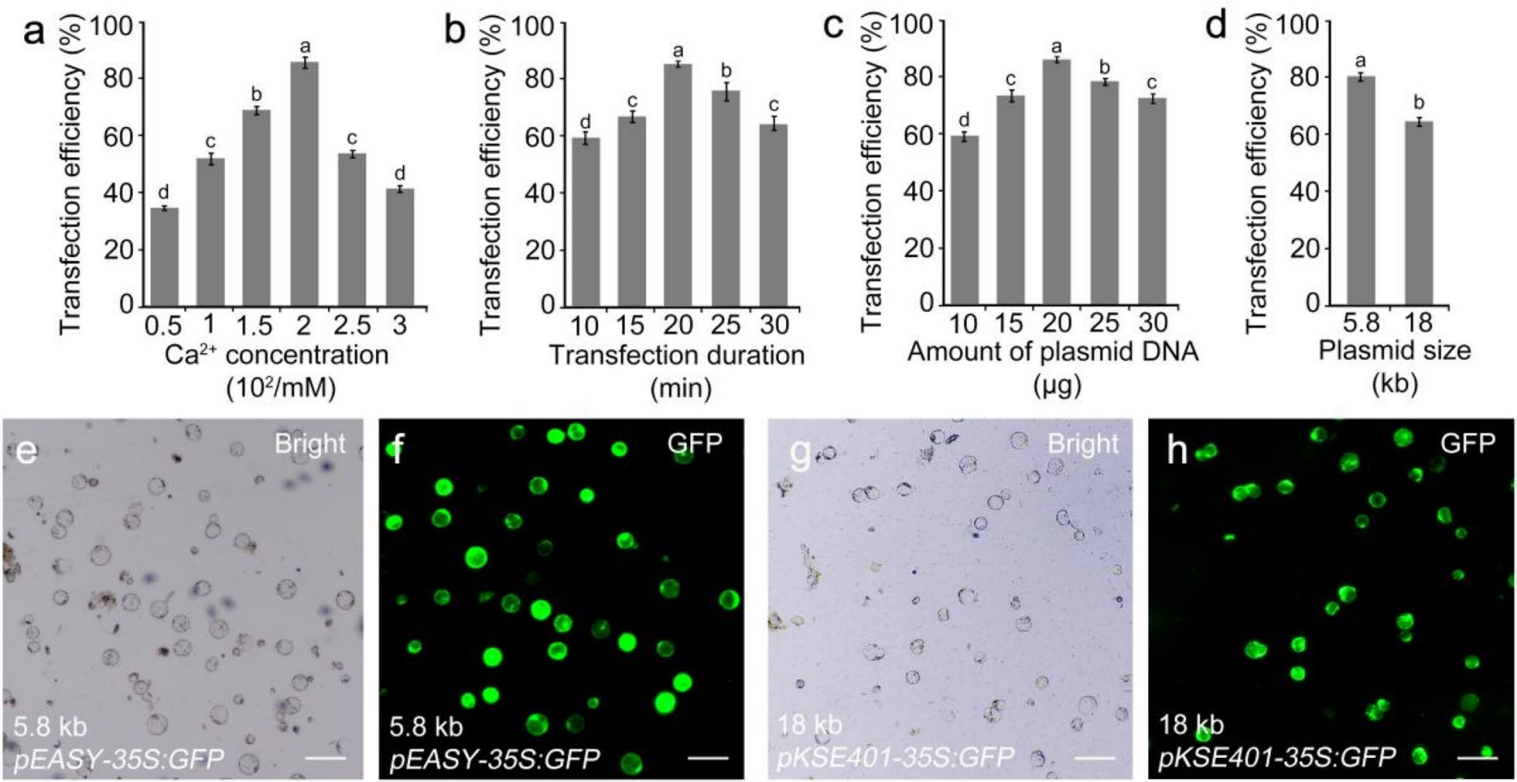
Optimization of the transient transformation system for cotton protoplasts. **a–d** Effects of Ca^2+^ concentration (**a**), transfection duration (**b**), plasmid concentration (**c**), and plasmid size (**d**) on transfection efficiency. **e** and **f** Bright field (**e**) and fluorescence microscopic (**f**) images of protoplasts transformed using *pEASY-35S:GFP* are shown. **g** and **h** Bright field (**g**) and fluorescence microscopic (**h**) images of protoplasts transformed using *pKSE401-35S:GFP* are shown

Thus, factors affecting PEG-mediated protoplast transfection (e.g., the concentration of Ca^2+^ in the PEG solution, incubation time, and plasmid concentration) were adjusted. The efficiency increased significantly from 31 to 84% by increasing the Ca^2+^ concentration from 50 to 200 mM, and then decreased to 40% by increasing the Ca^2+^ concentration to 300 mM (Fig. 3a). Moreover, the efficiency increased from 59 to 84% with an increase in incubation time from 10 to 20 min, respectively; however, it decreased to 64% with an incubation time of 30 min (Fig. 3b). Using 200 mM Ca^2+^ and 20 min of incubation, the transfection efficiency increased from 58 to 84% with an increase in plasmid concentration from 10 to 20 μg, and decreased to 71% with 30 μg of plasmid (Fig. 3c). Ultimately, our PEG-mediated protoplast transfection protocol was optimized as follows: roots, 72 h; Ca^2+^ concentration, 200 mM; incubation time, 20 min; and plasmid amount, 20 μg. By applying these parameters, a transfection efficiency of 84% was achieved (Fig. 3d–f). Moreover, 60% transfection efficiency was obtained using the large binary plasmid *pKSE401-35S:GFP* (Fig. 3d, g, h). This optimized system also worked well with other upland cotton varieties (e.g., R15) and island cotton Pima90-53 (*Gossypium barbadense*) (Additional file 6).

### Fast and efficient validation of CRISPR vectors using cotton protoplasts

**Fig. 4:**
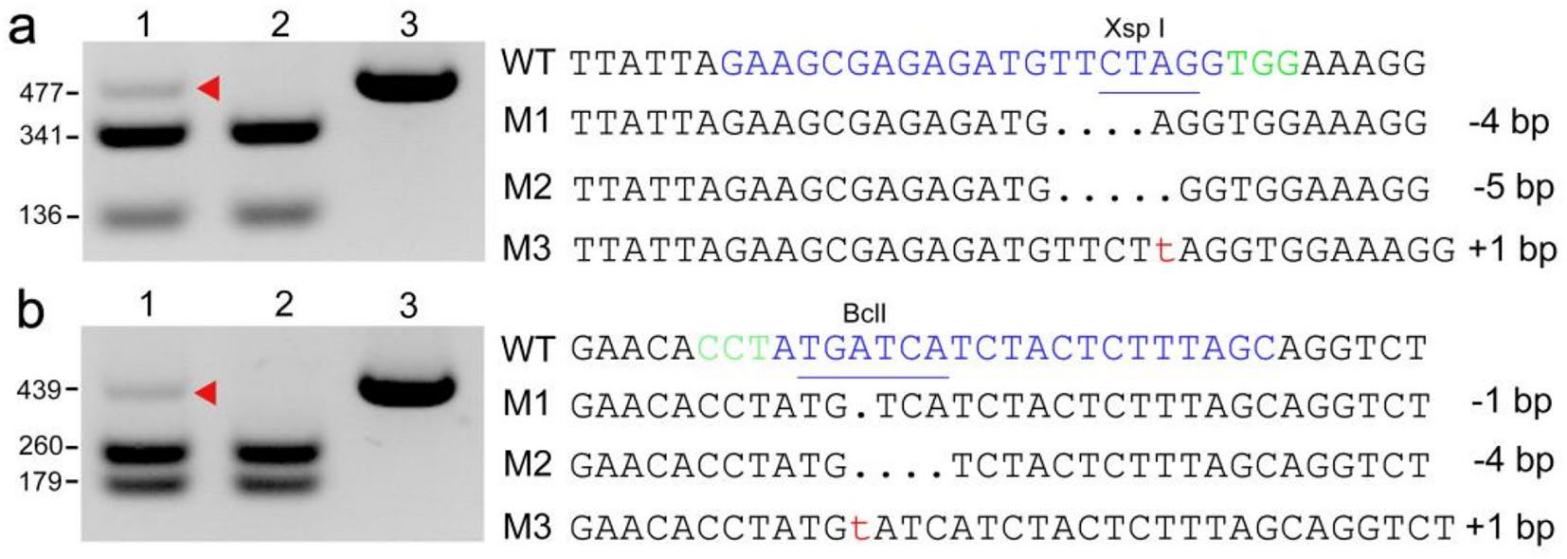
PCR/RE assays to detect CRISPR/Cas9-induced mutations in cotton protoplasts. **a** and **b** The activities of two sgRNAs were detected. Lane 1: digested PCR products amplified from samples treated with the respective sgRNAs. Lane 2: the digested wild-type control. Lane 3: the undigested wild-type control. Red arrowheads indicate bands with mutations. The sgRNA target sequence is in blue; the protospacer adjacent motif is in green

Stable cotton transformation methods are time- and labor-intensive [27]. Therefore, it is critical to develop an efficient transient method to verify the activity of CRISPR vectors in order to fully apply genome editing technologies to cotton. To test the effectiveness of transient PEG-mediated transformation, we used a previously designed single guide RNA (sgRNA), sgRNA1-*PDS* [27]. The activity of the resulting CRISPR vector was checked in cotton protoplasts using a PCR/RE assay. An uncleaved band was detected, isolated, and sequenced to confirm the expected CRISPR/Cas9-induced mutation (Fig. 4a). This transient gene expression system proved to be effective to detect target site activity.

We next tested our system using other genomic sites. To this end, we designed sgRNAs targeting *PDS* and *CLA*, respectively. After transforming the vectors into cotton protoplasts, PCR/RE assays were used and insertions/deletions induced by the sgRNA2-*PDS* were verified by sequencing (Fig. 4b). Thus, our system is broadly applicable and can be used to quickly validate the activity of CRISPR vectors.

### Protein subcellular localization in cotton protoplasts

**Fig. 5:**
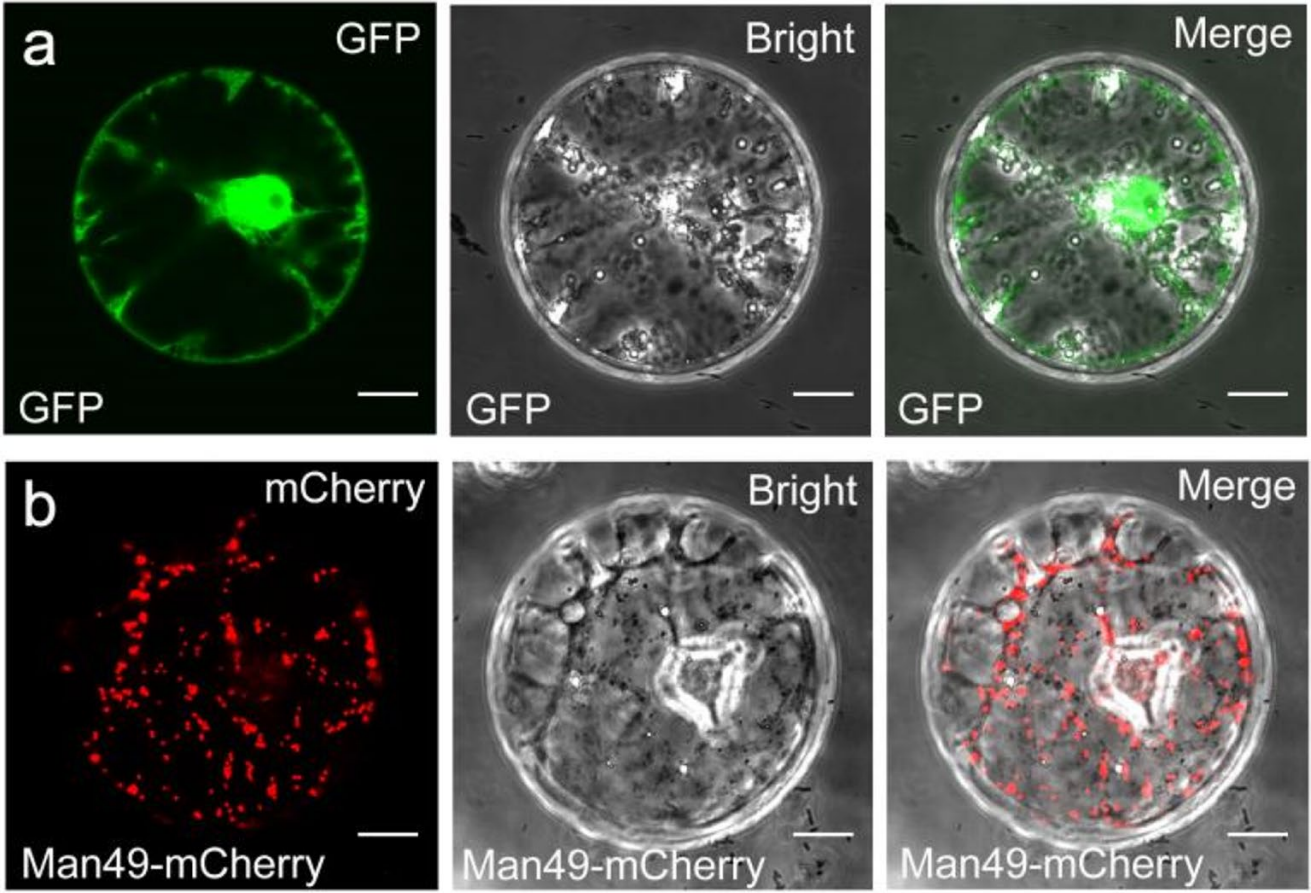
Protein subcellular localization studies of cotton root protoplasts. **a** and **b** *pEASY-35S:GFP* (GFP) or *pUC18-35S:Man49-mCherry* (Man49-mCherry) was transiently expressed in protoplasts derived from cotton roots. Merge, merged images of GFP or mCherry and bright field. Bars = 10 μm

Protein subcellular localization analysis based on protoplasts is an essential method to study protein function. Since chloroplasts are not present in root cells, the use of root cells for protein subcellular localization assays avoids interference from chloroplast autofluorescence [32]. To investigate the feasibility of using cotton root protoplasts for protein subcellular localization studies, the Golgi localization protein Man49 with an mCherry tag (*pUC18-35S:Man49-mCherry*) and GFP distributed ubiquitously in cell (*pEASY-35S:GFP*) were transformed into protoplasts [29]. Strong GFP fluorescence was noted in the nucleus, cell membrane, somewhere in the cytoplasm (Fig. 5a), while the fusion protein Man49-mCherry had a distinctive punctate distribution in the cells, which corresponded to the distribution pattern of the Golgi apparatus (Fig. 5b).

### Protein–protein interactions in cotton protoplasts

**Fig. 6:**
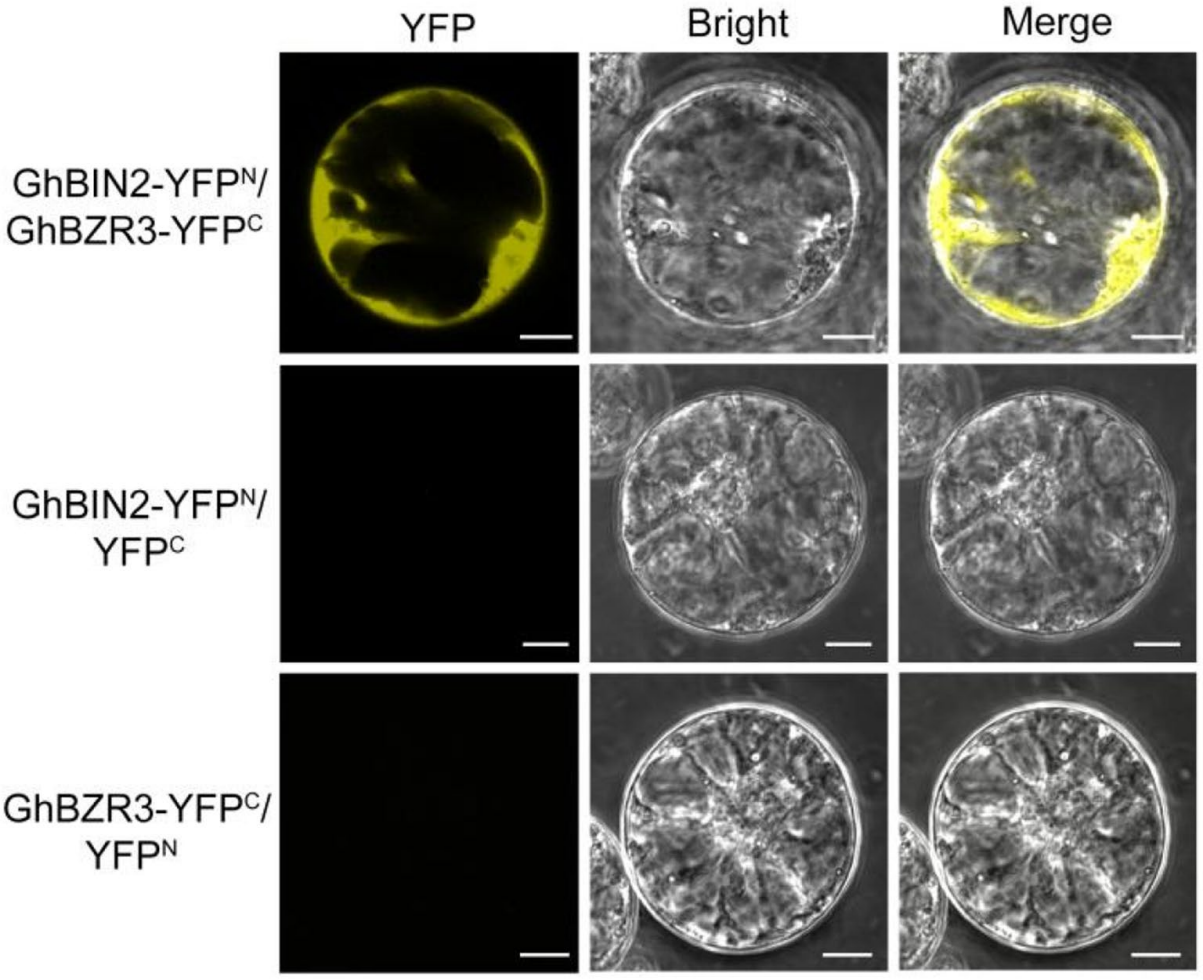
Protein–protein interaction assays in cotton root protoplasts. A BiFC analysis of the interaction between GhBIN2 and GhBZR3 was conducted in cotton root protoplasts. The paired constructs *p326-GhBIN2-YFP*^*N*^ (GhBIN2-YFP^N^) and p326-GhBZR3-YFP^C^ (GhBZR3-YFP^C^) were transiently co-expressed in protoplasts. GhBIN2-YFP^N^ or GhBZR3-YFP^C^ with the empty vector (p*326-YFP*^*C*^ or *p326-YFP*^*N*^) were used as negative controls. Merge, merged images of YFP and bright field. Bars = 10 μm

BiFC is widely used to detect protein–protein interactions. The GSK3-like kinase BRASSINOSTEROID (BR)-INSENSITIVE 2 (BIN2) and transcription factor BRASSINAZOLE RESISTANT 1 (BZR1) are well-known downstream components of the BR signal transduction pathway [34]. BIN2 interacts with and phosphorylates BZR1 or its homolog BES1 in *Arabidopsis*, leading to a change in localization of BZR1/BES1 from the nucleus to the cytoplasm [35]. This mechanism is conserved in other plants, including *Brassica rapa* [36]. We speculated that BIN2 and BZR3 also interact in cotton. Therefore, GhBIN2 (GhA09G0713) and GhBZR3 (Gh_A10G0312), which is localized mainly in the nucleus [37], were used as candidates in a BiFC assay in cotton protoplasts. YFP fluorescence representing GhBIN2-nYFP + GhBZR1-cYFP was observed in the cytoplasm, suggesting that BZR3 and BIN2 interacted with each other and that BZR3 was phosphorylated, triggering its export from the nucleus (Fig. 6). The negative controls, GhBIN2-YFP^N^ combined with empty YFP^C^(*p326-YFP*^*C*^) and empty YFP^N^ (*p326-YFP*^*N*^) combined with GhBZR3-YFP^C^, did not show YFP fluorescence. Thus, cotton protoplasts prepared using our method were suitable for the study of protein–protein interactions.

## Discussion

Cotton is of global economic importance; however, due to its polyploid nature and lengthy transformation procedure, few genes have been well studied [27]. Different protoplast isolation and transient expression protocols have been developed which facilitate functional genomics in numerous plants, including *Arabidopsis* [22], rice [23], and wheat [38]. However, few studies have reported protoplast isolation protocols and transient transformation systems for cotton [39, 40]. This may be because the high levels of polysaccharides and polyphenols in cotton leaves make protoplast isolation and transient expression more challenging than in other species. The protoplast isolation protocol and transient gene expression system based on true leaves and cotyledons have been established [39]. Meanwhile, Wang [40] reported the isolation of protoplasts from 3-month-old cotton calli. Although the yield and activity were suitable for subsequent experimental studies, preparing callus tissue is laborious and time-consuming. Here, we developed rapid and efficient protoplast isolation and transient expression protocols using cotton taproots, which has some unique advantages over other tissue protoplasts isolated methods For example, ①No disturbing. Chlorophyll in the leaf mesophyll cell is often associated with a high level of autofluorescence. It would be disturbed to use these cells for subcellular localization and other fluorescence-based analysis, especially when it comes to red fluorescent labels or dyes (for example, RFP, DsRed, mCherry, PI, and FM4-64); ②No gossypol contamination. The root of cotton did not contain gossypol, not like most cotton true leaves and cotyledons. The gossypol may be a kind of impurities to influence the activity of protoplasts, causing the browning reaction; ③High efficiency and time-saving. This root protoplasts isolated method is higher throughput and higher cell activities than before. The cotton taproots materials can be obtained in abundance in a shorter time, which are just grown for 72 h, while the cotyledons are grown for 12 days, and the fifth true leaves of cotton may cost more than one-month for material preparation [39].

The developmental stage of the materials used in protoplast isolation can greatly impact the outcome. For example, root age influences the quantity and quality of protoplasts. Liu [12] compared the yield and viability of protoplasts isolated from 5-, 7-, 9- and 10-day-old root tips. They found that 5-day-old root-generated protoplasts exhibited high viability (~ 85%) and were suitable for transcriptome sequencing. By comparing the quality and viability of protoplasts isolated from taproots of cotton grown in hydroponics for 48, 72, or 96 h, we found that the highest yield and viability of protoplasts were obtained from roots grown for 72 h. This is probably because the root tissue was youthful and tender, and the cell walls were thin, thus making protoplast isolation relatively easy [13, 14].

Although single-cell sequencing is a powerful technique for studying cellular responses to developmental and environmental cues, strict requirements must be met in terms of protoplast preparation [16]; otherwise, transcriptomic effects may be detected [16, 41]. Although it has been reported that the retention or elimination of induced genes has a limited impact on the clustering analysis of data and the identification of cell types, it has a significant impact on more intricate analyses, such as gene regulatory network analyses [42, 43]. To minimize transcriptomic effects, it is necessary to simplify the cell preparation process as much as possible; in particular, the duration of enzyme-induced hydrolysis should not exceed 3 h. To identify and filter genes induced during cell preparation, digested and unprocessed tissue can be compared in a sister RNA-seq experiment.

Cell size and viability also have important effects on the quality of scRNA-seq data. To avoid blocking the pipeline and to reduce size-biased effects, 10 × Genomics’ commonly used Chromium platform specifies that the cell size cannot exceed 40–50 μm [16]. Additionally, since the Ca^2+^ and Mg^2+^ contained in the buffer solution can interfere with subsequent reverse transcription and cause intercellular adhesion and clumping, protoplasts are generally resuspended in mannitol. We found that protoplasts should be resuspended in 0.5 M mannitol to maximize cell viability and reduce the production of LSCs. To further reduce the proportion of LSCs, it is necessary to filter the cells with a 30 μm cell strainer after using a 40 μm cell strainer. We also detected the effect of protoplast isolation on development-related genes regulated by epigenetic modifications and found that our protoplast isolation protocol had a slight effect on epigenetic remodeling.

Protoplast-based transient expression systems play important roles in plant functional genomics [14, 31, 40]. To obtain reliable experimental results, the protoplast transfection efficiency must be  > 50% [22]. Various factors influencing PEG-mediated protoplast transfection were optimized in this study. Highly efficient transfection (~ 80%) was achieved when protoplasts were isolated from cotton roots grown in hydroponics for 72 h, and when transfection was done using 20 μg of plasmid for 20 min in a PEG solution containing 200 mM Ca^2+^. Among these factors, we found that the condition of the roots was the most important factor influencing the transfection efficiency. Importantly, the transfection efficiency of large plasmids was ~ 60%, and the experimental results obtained were reproducible and reliable. Additionally, our optimized transient expression system worked well in other varieties and in island cotton. Thus, our protoplast-based transient expression system may be broadly applied to functional genomic studies of cotton.

CRISPR/Cas9-mediated genome editing is used to generate targeted mutants [18]. The application of CRISPR/Cas9 has changed the pace and course of plant science [5]; however, not all CRISPR vectors are active. Given that plant regeneration in cotton is time-consuming, it is critical to verify the activity of targets before stable transformation. Gao [27] established a transient expression system using cotton cotyledons. By infiltrating 10-day-old cotton cotyledons with *Agrobacterium* harboring CRISPR/Cas9 vectors, the efficiency of several target sites for endogenous genes was validated. To test the effectiveness of our established protoplast-based transient expression system, a previously reported site in *PDS* was chosen; the CRISPR-induced mutation was successfully detected by a PCR/RE assay and sequencing. Moreover, a new target site for *PDS* was verified and confirmed. However, one site targeting *CLA* had no significant editing efficiency. Thus, our work provides an effective strategy to quickly validate the target sites in CRISPR vectors, and it will promote the comprehensive application of genome editing technologies to cotton.

Transient protein expression is used for various applications in plant molecular genetic research. Subcellular localization is the most common type of experiment used to assess gene function. However, most cotton genes are transformed into *Arabidopsis* protoplasts, which may not be suitable given the different genetic backgrounds between cotton and *Arabidopsis*. Here, we transfected cotton protoplasts with a GFP-encoding vector, which is the most frequently used visual tag, and a Man49-mCherry-encoding vector, which is often used together with target genes as a marker to indicate the Golgi apparatus, and our experiments produced clear results. BiFC is a basic assay for demonstrating protein–protein interactions. Thus, our method provides a time-saving, efficient, and interference-free system for studies of gene function in cotton.

## Conclusions

We developed an efficient method for isolating cotton protoplasts from taproots. Moreover, we optimized a protoplast transient expression system to achieve a transfection efficiency of ~ 80%. The protocols developed here may be broadly applied to studies of cotton, including scRNA-seq, genome editing, protein localization, protein–protein interactions, and gene function identification.

## Supplementary Information

**Additional file 1:** Primers used in this study.

**Additional file 2:** Trypan blue staining of protoplasts from roots in different ages. **a–c **Protoplasts stained with Trypan blue, which were isolated from 48, 72, and 96 h roots. Bars=100 μm. **d** The effect of root age on protoplast viability.

**Additional file 3:** FDA staining of protoplasts from roots with different digestion time (1, 2, 3, 4, or 5 h). **a–e** Protoplasts stained with FDA, which were isolated from roots digested for different times. Bars=100 μm.

**Additional file 4:** Trypan blue staining of protoplasts resuspended with various concentrations of mannitol (0.3, 0.4, 0.5, 0.6, or 0.7 M). **a–e** Protoplasts stained with Trypan blue and resuspended in various concentrations of mannitol. Bars=100 μm.

**Additional file 5:** Initial transient transformation results of protoplasts. Protoplasts transformed with *pEASY-35S:GFP* were imaged under bright field, GFP, and merged channels, respectively. Bars=50 µm.

**Additional file 6:** Optimized transient transformation system of cotton protoplasts. **a–b** Transfection was conducted using protoplasts isolated from R15 and Pima90-53. Protoplasts transformed with *pEASY-35S:GFP* were imaged under bright field, GFP, and merged channels, respectively. Bars=50 µm.

## Data Availability

The datasets used and/or analyzed during the current study available from the corresponding author on reasonable request.
