# Supplementary material for: Establishment of an efficient cotton root protoplast isolation protocol suitable for single-cell RNA sequencing and transient gene expression analysis
Source: Plant Methods. 2023 Jan 18;19:5. doi: 10.1186/s13007-023-00983-6 (PMC9850602; doi:10.1186/s13007-023-00983-6)
Supplement: Supplementary file 1 — Additional file 1: Primers used in this study. [file 13007_2023_983_MOESM1_ESM.docx]

**Additional files**

**Additional file 1.** Primers used in this study.

| Primer name | Sequence（5’–3’） | Function |
| --- | --- | --- |
| sgRNA1-PDS-F | GGCGGAAGCGAGAGATGTTCTAGG | Constructing sgRNA1-PDS CRISPR vector |
| sgRNA1-PDS-R | AAACCCTAGAACATCTCTCGCTTC |  |
| sgRNA2-PDS-F | GGCGGCTAAAGAGTAGATGATCAT | Constructing sgRNA2-PDS CRISPR vector |
| sgRNA2-PDS-R | AAACATGATCATCTACTCTTTAGC |  |
| sgRNA1-CLA-F | GGCGCATATCAGAATCAAGATATC | Constructing sgRNA1-CLA CRISPR vector |
| sgRNA1-CLA-R | AAACGATATCTTGATTCTGATATG |  |
| PDS-1F | catcactcaagtttgtttttgt | Detecting activity of sgRNA1-PDS |
| PDS-1R | GAACGAAAGGCCCTTTCTTTC |  |
| PDS-2F | TGAATTATTACTTGCTAGGGCT | Detecting activity of sgRNA2-PDS |
| PDS-2R | GCAGAGGACCTAGAATGGTAC |  |
| CLA-1F | cttttcccttgccttctaga | Detecting activity of sgRNA1-CLA |
| CLA-1R | CTTGGTAACTCCctgcaata |  |
| GhHDA6-F | CTTATGCGAACAAGCACCAAA | Realtime PCR |
| GhHDA6-R | TTCAAACGACGGACTCTACCC |  |
| GhSWN-F | CTGCTGGGCGTGAATGTGA | Realtime PCR |
| GhSWN-R | TCCCTCGTTTGTCTGCTTCTG |  |
| GhJMJ25-F | CCACGTCATCATGCCGAGTT | Realtime PCR |
| GhJMJ25-R | TGAGTCTCCACGCCCAAGC |  |
| GhACTIN14-F | ATCCTCCGTCTTGACCTTG | Realtime PCR |
| GhACTIN14-R | TGTCCGTCAGGCAACTCAT |  |
| GhBIN2-F | AATTAATTAACATGACTAGTATGGCTGAAAATAAGGAAAT | Constructing GhBIN2-YFP^N^ BiFC vector |
| GhBIN2-R | CTGCCACCTCCTCCACTAGTTGTTCTGGCTGGATGCATGA |  |
| GhBZR3-F | AATTAATTAACATGACTAGTATGACGTCAGGGACGAGAAT | Constructing GhBZR3-YFP^C^ BiFC vector |
| GhBZR3-R | CTGCCACCTCCTCCACTAGTCCTGGTTTTTGAATTTCCAA |  |
