# Supplementary material for: Establishment of an efficient cotton root protoplast isolation protocol suitable for single-cell RNA sequencing and transient gene expression analysis
Source: Plant Methods. 2023 Jan 18;19:5. doi: 10.1186/s13007-023-00983-6 (PMC9850602; doi:10.1186/s13007-023-00983-6)
Supplement: Supplementary file 2 — Additional file 2: Trypan blue staining of protoplasts from roots in different ages. a–c Protoplasts stained with Trypan blue, which were isolated from 48, 72, and 96 h roots. Bars=100 μm. d The effect of root age on protoplast viability. [file 13007_2023_983_MOESM2_ESM.docx]

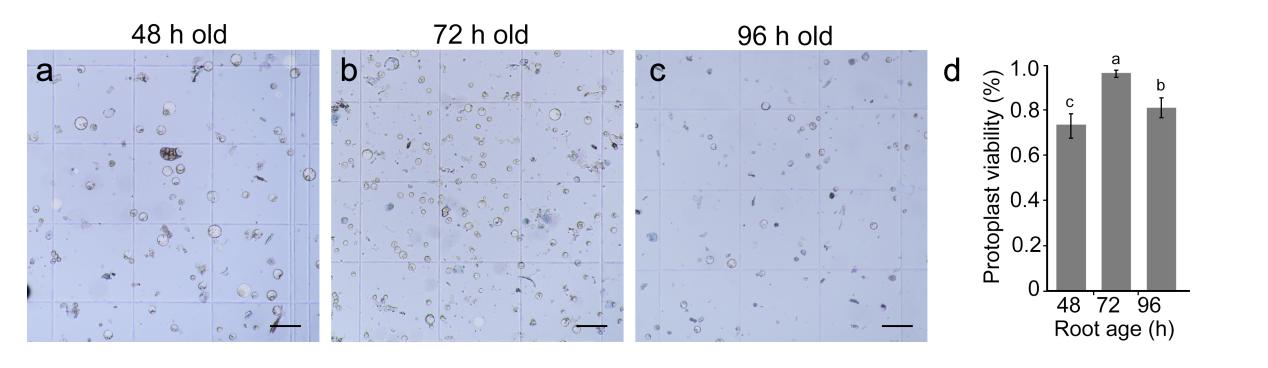


**Additional file 2.** Trypan blue staining of protoplasts from roots in different ages. **a–c** Protoplasts stained with Trypan blue, which were isolated from 48, 72, and 96 h roots. Bars=100 μm. **d** The effect of root age on protoplast viability.
