# Supplementary material for: Establishment of an efficient cotton root protoplast isolation protocol suitable for single-cell RNA sequencing and transient gene expression analysis
Source: Plant Methods. 2023 Jan 18;19:5. doi: 10.1186/s13007-023-00983-6 (PMC9850602; doi:10.1186/s13007-023-00983-6)
Supplement: Supplementary file 3 — Additional file 3: FDA staining of protoplasts from roots with different digestion time (1, 2, 3, 4, or 5 h). a–e Protoplasts stained with FDA, which were isolated from roots digested for different times. Bars=100 μm. [file 13007_2023_983_MOESM3_ESM.docx]

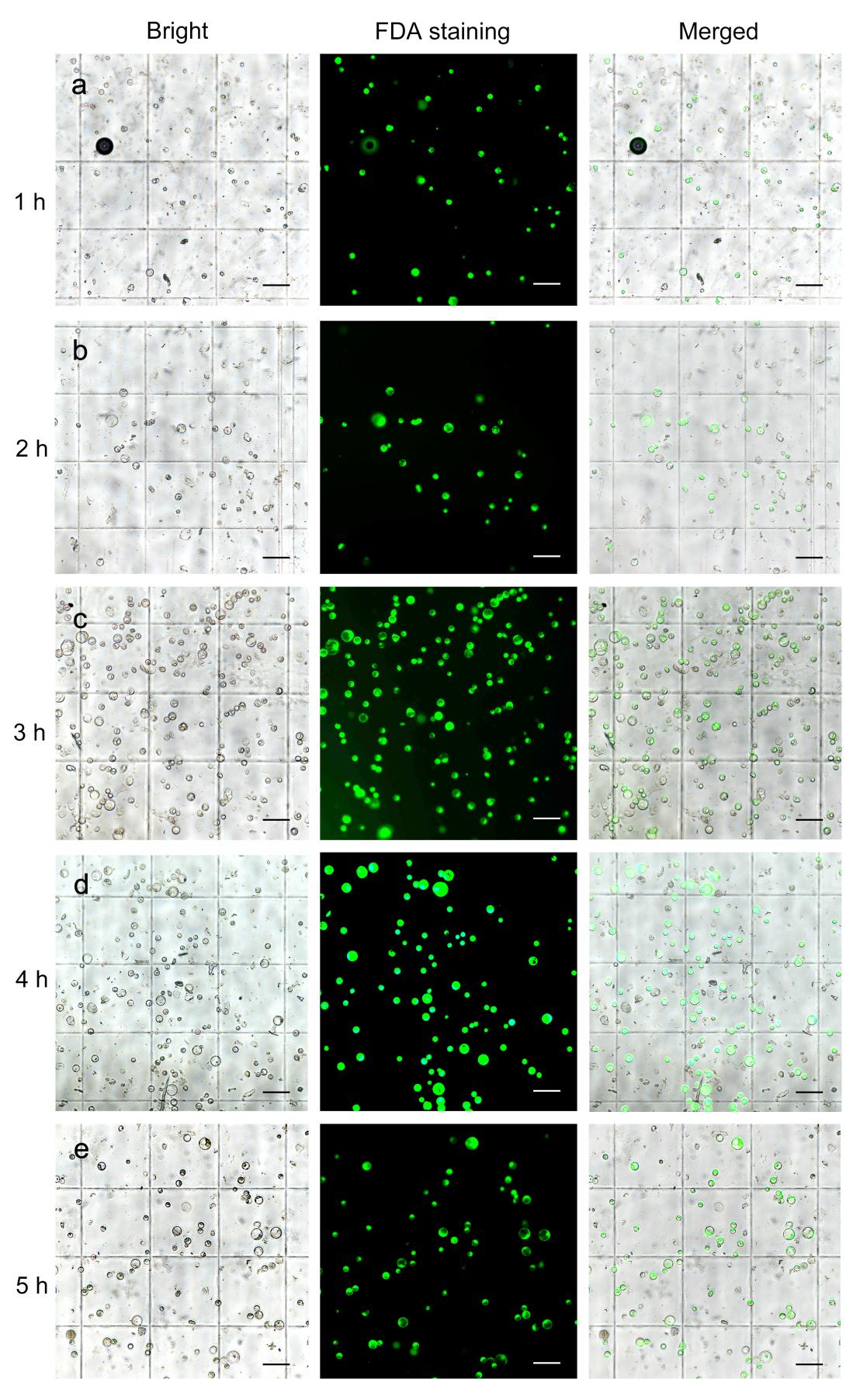


**Additional file 3.** FDA staining of protoplasts from roots with different digestion time (1, 2, 3, 4, or 5 h). **a–e** Protoplasts stained with FDA, which were isolated from roots digested for different times. Bars=100 μm.
