# Supplementary material for: Establishment of an efficient cotton root protoplast isolation protocol suitable for single-cell RNA sequencing and transient gene expression analysis
Source: Plant Methods. 2023 Jan 18;19:5. doi: 10.1186/s13007-023-00983-6 (PMC9850602; doi:10.1186/s13007-023-00983-6)
Supplement: Supplementary file 4 — Additional file 4: Trypan blue staining of protoplasts resuspended with various concentrations of mannitol (0.3, 0.4, 0.5, 0.6, or 0.7 M). a–e Protoplasts stained with Trypan blue and resuspended in various concentrations of mannitol. Bars=100 μm. [file 13007_2023_983_MOESM4_ESM.docx]

**
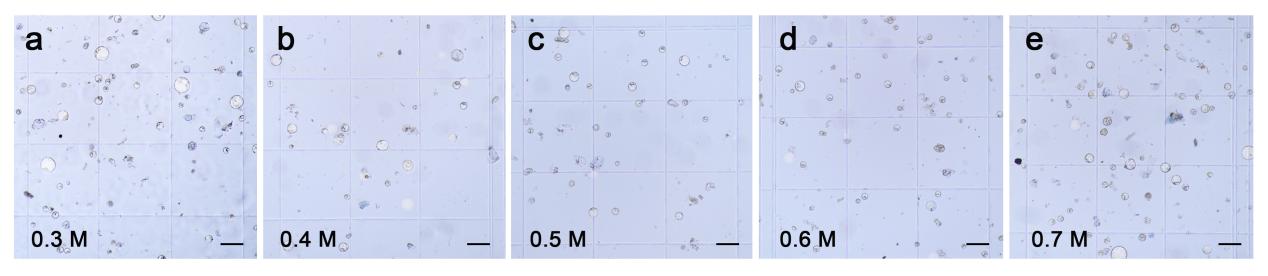
Additional file 4.** Trypan blue staining of protoplasts resuspended with various concentrations of mannitol (0.3, 0.4, 0.5, 0.6, or 0.7 M). **a–e** Protoplasts stained with Trypan blue and resuspended in various concentrations of mannitol. Bars=100 μm.
