# Supplementary material for: Establishment of an efficient cotton root protoplast isolation protocol suitable for single-cell RNA sequencing and transient gene expression analysis
Source: Plant Methods. 2023 Jan 18;19:5. doi: 10.1186/s13007-023-00983-6 (PMC9850602; doi:10.1186/s13007-023-00983-6)
Supplement: Supplementary file 6 — Additional file 6: Optimized transient transformation system of cotton protoplasts. a–b Transfection was conducted using protoplasts isolated from R15 and Pima90-53. Protoplasts transformed with pEASY-35S:GFP were imaged under bright field, GFP, and merged channels, respectively. Bars=50 µm. [file 13007_2023_983_MOESM6_ESM.docx]

**
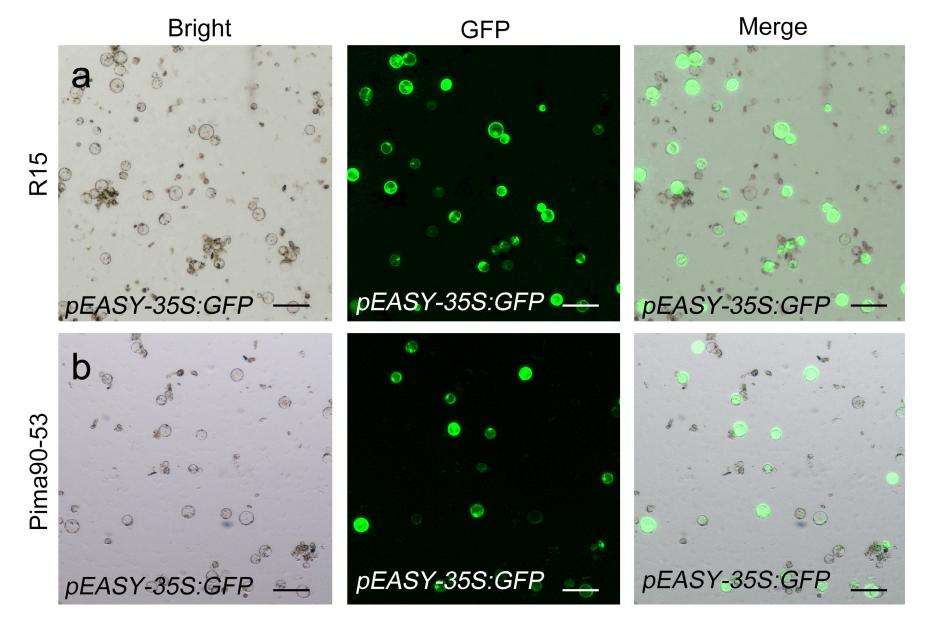
**

**Additional file 6.** Optimized transient transformation system of cotton protoplasts. **a–b** Transfection was conducted using protoplasts isolated from R15 and Pima90-53. Protoplasts transformed with *pEASY-35S:GFP* were imaged under bright field, GFP, and merged channels, respectively. Bars=50 µm.
